# Glycogen Synthase Kinase 3 (GSK3) Inhibitor, SB-216763, Promotes Pluripotency in Mouse Embryonic Stem Cells

**DOI:** 10.1371/journal.pone.0039329

**Published:** 2012-06-26

**Authors:** Leslie A. Kirby, Jason T. Schott, Brenda L. Noble, Daniel C. Mendez, Paul S. Caseley, Sarah C. Peterson, Tyler J. Routledge, Nilay V. Patel

**Affiliations:** 1 Department of Biological Science, California State University – Fullerton, Fullerton, California, United States of America; 2 Center for Applied Biotechnology Studies, California State University – Fullerton, Fullerton, California, United States of America; Baylor College of Medicine, United States of America

## Abstract

Canonical Wnt/β-catenin signaling has been suggested to promote self-renewal of pluripotent mouse and human embryonic stem cells. Here, we show that SB-216763, a glycogen synthase kinase-3 (GSK3) inhibitor, can maintain mouse embryonic stem cells (mESCs) in a pluripotent state in the absence of exogenous leukemia inhibitory factor (LIF) when cultured on mouse embryonic fibroblasts (MEFs). MESCs maintained with SB-216763 for one month were morphologically indistinguishable from LIF-treated mESCs and expressed pluripotent-specific genes Oct4, Sox2, and Nanog. Furthermore, Nanog immunostaining was more homogenous in SB-216763-treated colonies compared to LIF. Embryoid bodies (EBs) prepared from these mESCs expressed early-stage markers for all three germ layers, and could efficiently differentiate into cardiac-like cells and MAP2-immunoreactive neurons. To our knowledge, SB-216763 is the first GSK3 inhibitor that can promote self-renewal of mESC co-cultured with MEFs for more than two months.

## Introduction

Pluripotent embryonic stem cells (ESCs) have potential use in regenerative medicine because they can differentiate into cell types from all three germ layers. The quality of ESCs is critical for their long-term maintenance and efficient differentiation into desired lineages. Thus, identification of novel drugs that promote ESC pluripotency and self-renewal is of great interest to the fields of stem cell biology and regenerative medicine [1]–[3]. The classic method for maintenance of mouse ESC (mESC) pluripotency is by supplementation of medium with leukemia inhibitory factor (LIF) [4]–[6]. In contrast to mESCs, most human ESC (hESC) lines are thought to be epiblast-like and require basic fibroblast growth factor-2 (FGF2) to retain their pluripotent state [3], [7]. However, reprogramming of human fibroblasts into induced pluripotent stem cells in the presence of LIF yields hESCs that are LIF-dependent and comparable to ground state mESCs [8]. LIF maintains pluripotency in stem cells by parallel induction of Sox2 and Nanog through Klf4 and Tbx3, respectively, and downstream activation of Oct4 [9]. Activation of the “trinity” factors – Oct4, Sox2 and Nanog – is central to maintenance of pluripotency [3], [7], [9]–[12]. However, other signal transduction pathways, including the canonical Wnt pathway, can also activate these core pluripotency-enabling transcription factors [1]–[3], [13], [14].

Wnt pathways are critical for developmental processes including self-renewal, cell proliferation, lineage specification, and maintenance of adult stem cells in a multipotent state [15]–[18]. The canonical Wnt pathway is activated when canonical Wnt glycoproteins bind a membrane-associated receptor complex containing frizzled (Fzd) and LRP family receptors [18]–[21]. Wnt binding activates Disheveled (Dvl), which then inhibits a second complex known as the β-catenin degradation complex [20], [21]. The β-catenin degradation complex is comprised of axin, glycogen synthase kinase-3 (GSK3), and adenomatous polyposis coli (APC) [20], [21]. Normally, the transcriptional co-activator β-catenin is recruited to the axin-GSK3 complex by APC, where β-catenin is phosphorylated by GSK3 and targeted for proteosomal degradation [20]–[22]. Both isoforms of GSK3– GSK3α and GSK3β – are able to form a complex with APC and axin to phosphorylate β-catenin [23]–[25]. When the canonical Wnt pathway is activated, β-catenin can translocate to the nucleus to promote gene expression through transcription factor/lymphoid enhancer-binding factor (TCF/LEF) response elements [17], [18], [21]. When β-catenin is not present in the nucleus, TCF3 bound to TCF/LEF sites inhibits expression of various pluripotency target genes through its interactions with Oct4, Sox2, and Nanog transcription factors [26], [27]. When β-catenin does become available in the nucleus, it binds to TCF3 and alleviates TCF3’s repression of pluripotency promoting genes [26]–[32]. This β-catenin mediated expression of pluripotency genes is further fine-tuned by co-regulators, other TCFs, and the trinity transcription factors [14], [27], [33].

Another direct target of the β-catenin/TCF3 heterodimer is the orphan nuclear receptor Lrh-1 [13]. β-catenin-deficient mESCs have lower expression of Lrh-1, Oct4 and Nanog, and overexpression of Lrh-1 in these mESCs restores expression of Oct4 and Nanog [13]. The existence of the Lrh-1 pathway further supports the idea that β-catenin is critical for pluripotency. Consistent with this interpretation, mESCs lacking both isoforms of GSK3 express Oct4 and Nanog for an extended period of time and fail to differentiate [25], [32], [33]. Despite the strong genetic evidence in support of β-catenin’s role, no known pharmacological agent that can stabilize β-catenin has been shown to maintain pluripotency by itself for more than a week [1], [2], [33]–[36]. Here, we report that 6-bromoindirubin-3′-oxime (BIO), CHIR-99021, and SB-216763 effectively activate β-catenin mediated transcription, but only SB-216763 can maintain mESCs co-cultured with MEFs in a pluripotent state in the absence of exogenous LIF for up to two months.

## Materials and Methods

### Cell Culture

CF-1 mouse embryonic fibroblasts (MEF; SCRC-1040, ATCC) were used as a feeder layer for mESC cell culture. J1 mESC (SCRC-1010, ATCC) and MilliTrace Nanog GFP Reporter mESC (SCR089, Millipore) cell lines were cultured on MEFs that were mitotically inactivated using 10 µg/mL mitomycin C (Sigma). MESCs were cultured in DMEM (Mediatech) supplemented with 1.0 mM non-essential amino acids (NEAA), 0.1 mM β-mercaptoethanol (Sigma), 1% penicillin/streptomycin (MP Biomedicals), and 15% ESC qualified fetal bovine serum (ES-FBS; SCRR-30-2020, ATCC). As indicated, the medium also contained 1,000 U/mL mouse LIF (ESG1107, Chemicon), various concentrations of SB-216763 (BIOMOL International), BIO (Cayman Chemical), and CHIR-99021 (Cayman Chemical) dissolved in 0.1% DMSO, or no supplements [referred to as LIF-free or LIF (-)]. Cells were incubated at 37°C with 5% CO_2_ in a Sanyo MCO18AICUV cell culture incubator. Media were exchanged every two days. The mESC cultures were passaged every 3–4 days at densities needed to yield 5% confluent mESC cultures on the day of passaging (generally, between 1∶4 and 1∶8 dilutions). To minimize the carry-over of MEFs, the cultures were treated with 0.25% trypsin for 50 seconds in the incubator. Trypsin was quenched with ES-FBS-containing medium (without any supplements) and removed from the sample by centrifugation and resuspension of mESCs in the treatment-containing medium.

HEK293 cells (human embryonic kidney cell line; CRL-1573, ATCC) were cultured in DMEM supplemented with 1% penicillin/streptomycin and 10% bovine growth serum (HyClone). Cells were incubated at 37°C with 5% CO_2._ Cultures were passaged every 3 days.

The cells were monitored using either an Olympus CKX41 inverted microscope with stop-contrast objectives or an Olympus IX51 inverted microscope equipped with Hoffman Modulation Contrast system. Optical sectioning provided by the Hoffman Modulation Contrast system eliminated the “halo” around the mESC colonies. Pictures were captured using a digital, color CCD Infinity2 camera and the software provided by the manufacturer (Lumenera).

### Super TOPFlash Reporter Assay

J1 mESCs were electroporated with reporter genes using the NEON Transfection System according to the manufacturer’s protocol (Life Technologies). The day before electroporation of J1 mESCs, mitomycin C inactivated MEFs were plated at 12,000 cells/well in white, clear-bottom 96-well assay plates (Costar 3610). The following day, 1×10^6^ J1 mESCs were electroporated with 4.5 µg of Super TOPFlash (a β-catenin reporter with 7 concatenated TCF/LEF response elements upstream of firefly luciferase [37]; Addgene plasmid 12456), and 0.5 µg of pGL4.73 *Renilla* luciferase control reporter (Promega) in 100 µL NEON tips using a 1400 v, 10 ms pulse width, pulse number 3 setting. Electroporated J1 cells were plated on the MEFs at 5,000 cells/well in antibiotic-free mESC media supplemented with LIF. The next day, the mESC-MEF co-cultures were treated with vehicle (0.1% DMSO) or various doses of LIF, SB-216763, BIO, and CHIR-99021 in LIF-free mESC medium. The cells were lysed 24 hours later with Passive Lysis Buffer (Promega) and reporter activity was quantified using the Dual Luciferase Assay (Promega) in a GLOMAX 96 Microplate Luminometer equipped with dual injectors (Promega). The firefly data were normalized to *Renilla* luciferase activity and then to the vehicle control.

HEK293 cells were also transfected using the NEON Transfection System. Approximately 5×10^6^ HEK293 cells were electroporated with 9 µg of Super TOPFlash and 1 µg of pGL4.73 in 100 µL NEON tips using a 1100 v, 20 ms pulse width, pulse number 2 setting. Electroporated cells were diluted in antibiotic-free HEK293 medium and then plated at 20,000 cells/well in 96-well plates (Costar 3610). After 24 hours, treatments, cell lysis, and luciferase quantification were carried out as described for J1 mESCs, with the exception that all treatments were performed in serum-free Opti-MEM.

### MESC Pluripotency Assay

J1 mESCs or MilliTrace Nanog GFP Reporter mESCs were passaged in 6-well plates as described above. LIF-free mESC medium was supplemented with the indicated treatment immediately prior to culturing. MESCs for all conditions originated from cultures maintained with LIF. Passaging occurred in parallel for all treatments at regular intervals as determined by the fastest growing culture. Subcultures were prepared by seeding 20,000–50,000 cells/well in 6-well plates. The morphology of the J1 mESC cultures was monitored two days after passaging. Individual colonies were then rated according to the degree of compactness, circular shape, and the size of individual cells within the colony. The number of total colonies and pluripotent-like colonies were counted in five non-overlapping fields encompassing more than 25% of the well area (from one edge of the well to the opposite edge). The J1 mESCs maintained with the indicated supplements were (a) immunostained for Nanog and Oct4, (b) stained for Alkaline Phosphatase activity according to manufacturer’s protocol (AP Staining Kit II, Stemgent), (c) assayed for expression of pluripotency markers by quantitative PCR, or (d) differentiated into cardiac-like cells and neurons as described below.

For the MilliTrace Nanog GFP Reporter mESCs, forty colonies were scored based on the intensity and homogeneity of GFP expression in each colony. Briefly, the highest score was assigned to colonies that were thoroughly and intensely fluorescent. The colonies that had incomplete labeling were scored based on the approximate percentage of the colony that was intensely fluorescent. Because MilliTrace Nanog GFP Reporter mESCs continued to express low levels of GFP after one month of culture in LIF-free conditions, the score for the LIF-free samples was subtracted from the score for all samples.

### Neuronal Differentiation

MESCs maintained with LIF or 10 µM SB-216763 for more than a month were directed to differentiate into neurons using the 4−/4+ protocol [38]. MESCs were grown in 0.1% gelatin-coated 6-well plates in the indicated medium for 2 days. The mESC-enriched cultures were then trypsinized and two million mESCs were plated on Petri dishes in embyroid bodies (EB) formation medium (DMEM supplemented with 1.0 mM NEAA, 0.1 mM β-mercaptoethanol, 1% penicillin/streptomycin, and 10% ES-FBS). Free-floating EBs were incubated 4 days with a single exchange of medium after 2 days. The medium was then exchanged with EB formation medium supplemented with 0.5 µM all-trans retinoic acid (atRA; BIOMOL International) and EBs were incubated for 4 more days with a single exchange of medium after 2 days. These induced neural precursor cells were seeded on a tissue culture plate coated with poly-L-ornithine (MP Biomedicals) and laminin (Sigma) in EB formation medium without atRA for 2 weeks. Medium was exchanged every other day during this period. The cultures were then immunostained for MAP-2.

### Cardiac-like Differentiation

MESCs maintained with LIF or 10 µM SB-216763 for more than a month were resuspended at 40,000 cells/mL in LIF-free mESC medium. EBs were prepared by a hanging drop procedure. Briefly, 20 µL drops containing mESCs were pipetted on the inside of a 10-cm Petri dish lid. The lids were placed onto Petri dishes containing 10 mL of HBSS and the EBs were allowed to form and grow for 4 days in the incubator. After 4 days, 15–20 EBs were transferred to a well containing LIF-free mESC medium in a 24-well plate. The medium was exchanged every two days and autonomously beating cell aggregates were observed and counted.

### Immunocytochemistry

Cells were fixed with cold 4% paraformaldehyde in PBS for 15 minutes at room temperature and then permeabilized with 0.1% Triton-X 100 in PBS for 30 minutes. Serum blocking of non-specific epitopes was carried out with 1% normal goat serum in 0.1% Triton-X 100/PBS for 30 minutes. Samples were incubated with primary antibodies overnight at 4°C and then washed 5 times with PBS for 5 minutes each. Secondary antibody was incubated for 1 hour at room temperature, followed by PBS washes as described above. MESC pluripotency was assessed with antibodies for pluripotency-specific markers Nanog (Abcam; ab21603; 1∶500) or Oct–4 (Abcam ab19857; 1∶1000). Neuronal differentiation was confirmed by staining for the neuronal-specific marker MAP-2 (Santa Cruz Biotechnology; sc-20172; 1∶1000). Alexa Fluor 488 goat anti-rabbit IgG was used as the secondary antibody against all the primary antibodies (Life Technologies; A11008, 1∶500). Fluorescent images were captured with an Olympus IX51 inverted microscope equipped with Infinity2 CCD camera system or a Leica Microsystems inverted confocal microscope.

### Quantitative PCR for Pluripotency-specific Genes and Germ Line Markers

Total RNA was extracted from all samples using TRIzol reagent (Life Technologies). RNA was extracted from mESCs that were continuously cultured with LIF or 10 µM SB-216763 for one month. RNA from EBs was isolated 4 days after EB formation. RNA from cardiac-like cells was extracted six days after EBs were seeded on tissue culture dishes (when the number of autonomously contracting clusters had peaked). RNA from cultures directed to differentiate into neurons was extracted four days after atRA was added to the cultures.

First strand cDNA was synthesized with MMLV reverse transcriptase (Promega). Quantitative PCR was carried out using SYBR Green containing SensiMix (Bioline) and CFX96 Real-Time PCR detection system (BioRad). Assay conditions were as follows: 10 minute initial denaturation at 95°C and 40 cycles of 15 seconds at 95°C, 15 seconds at 60°C and 15 seconds at 72°C. The PCR mix contained 250 nM of each primer (Table S1) and 5–-30 ng of cDNA. The reactions were performed in triplicates and the results were analyzed using qbasePLUS (Biogazelle) with β-actin as the housekeeping gene.

### Statistical Analysis

For the morphology-based evaluation of J1 mESCs maintained in various treatments for over a month, the total number of colonies and the total number of pluripotent-like colonies were quantified in five non-overlapping fields. Average data for samples were analyzed with GraphPad Prism 5 software using one-way ANOVA and Dunnett post-hoc analysis for comparing LIF-treated mESCs with the remaining treatments. For MilliTrace Nanog GFP Reporter mESCs, data from two independent experiments was analyzed as described above for J1 mESCs.

## Results

### SB-216763 Induces β-catenin Mediated-transcription in a Dose-dependent Manner

**Figure 1:**
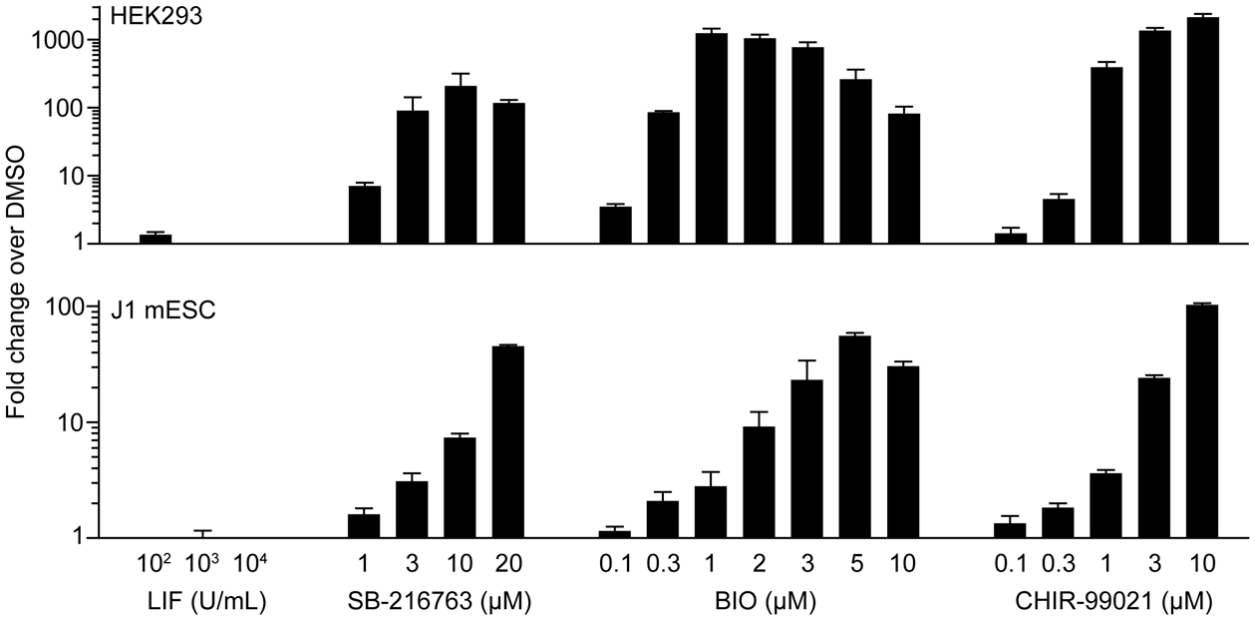
SB-216763, BIO and CHIR-99021 induce expression of a β-catenin reporter gene in a dose dependent manner. HEK293 cells (top panel) and J1 mESCs (bottom panel) were electroporated with the Super TOPFlash and pGL4.73 *Renilla* luciferase reporters. The cells were treated in quadruplicate wells with a vehicle control (0.1% DMSO) or the indicated doses of LIF, SB-216763, BIO, or CHIR-99021 for 24 hours. Cell lysates were then assayed for dual luciferase reporter activity. Firefly luciferase activity was normalized to *Renilla* luciferase activity for each sample, and then all results were normalized to the vehicle control. Data are expressed as the mean ± SEM from three independent trials.

SB-216763 is a potent inhibitor of both GSK3α and GSK3β, and can induce robust expression of a β-catenin reporter gene in HEK293 cells [39]–[41]. As expected, HEK293 cells treated with 1–20 µM SB-216763 exhibited a dose-dependent increase in Super TOPFlash activity (Figure 1; top). A similar TOPFlash dose-response for SB-216763 was also observed in J1 mESCs co-cultured with MEFs (Figure 1; bottom). BIO induced maximal activation of TOPFlash activity at a dose of 1 µM in HEK293 cells (1230-fold) and 5 µM in J1 mESCs (55-fold). The reduction in TOPFlash activation observed for 3–10 µM BIO in HEK293 cells is very likely associated with the toxic effects we observed in the serum-free Opti-MEM treatment media; 10 µM BIO also appeared to reduce the proliferation of J1 mESCs, resulting in decreased reporter activity. CHIR-99021 was the most potent inducer of TOPFlash activity in HEK293 cells (2130-fold at 10 µM) and J1 mESCs (103-fold at 10 µM).) Two µM BIO and 3 µM CHIR-99021 have been previously tested in mESC pluripotency assays by other groups [34], [35]. Our results show that the activation of TOPFlash by 2 µM BIO (9.2-fold) and 3 µM CHIR-99021 (24-fold) is comparable to activation by 10–20 µM SB-216763 (7.3- and 45-fold, respectively).

Comparison of the two panels in Figure 1 suggests that the J1 mESCs were less responsive to the GSK3 inhibitor treatments. However, the results for J1 mESCs and HEK293 cannot be directly compared because the J1 mESCs were treated in the presence of 15% ES-FBS, whereas HEK293 cells were treated in serum-free Opti-MEM. We performed the TOPFlash assay in J1 mESCs in serum-containing medium to allow for an accurate assessment of the level of β-catenin activation observed under normal mESC culture conditions. Furthermore, MEFs are also known to secrete Wnt proteins, which could activate β-catenin in mESCs [34], [42]. This would increase reporter activity in the vehicle treated samples and therefore proportionally reduce the fold-activations in the treated samples. Consistent with previous results, we also show that 100–10,000 U/ml of LIF does not further activate the TOPFlash reporter in J1 mESCs [43]. Specifically, this suggests that the LIF supplement does not induce secretion of canonical Wnt proteins from mESCs or MEFs.

### SB-216763 Maintains mESCs with a Pluripotent-like Morphology

**Figure 2:**
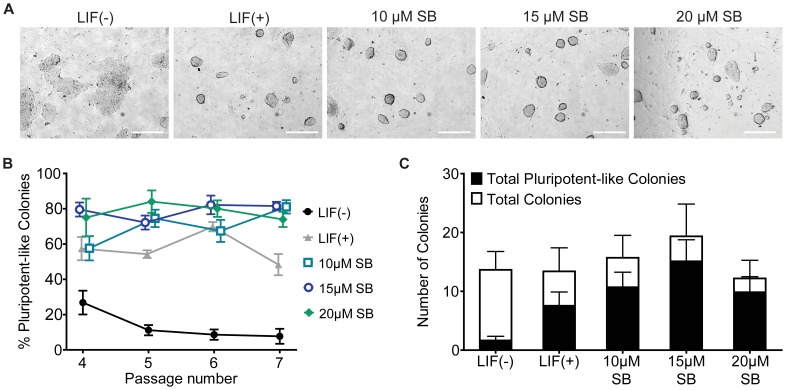
J1 mESCs maintained with SB-216763 have a pluripotent-like morphology. (A) Phase-contrast images of mESCs treated with LIF (1,000 U/mL), or 10, 15, and 20 µM SB-216763 for more than a month. All doses of SB-216763 maintained mESCs as compact, elliptical or circular colonies characteristic of pluripotent cells. Morphology for mESC colonies maintained with SB-216763 was comparable to those in LIF supplemented medium. Scale bars represent 100 µm. (B) Twenty thousand mESCs from each treatment were seeded in a 6-well chamber at each passage and monitored for the percentage of pluripotent-like colonies two days after each passage. Data are expressed as the mean of five fields analyzed ± SEM. The percentage of pluripotent-like colonies in vehicle-treated cultures was significantly different from 15 µM and 20 µM SB-216763 samples at passage 4 (p<0.05) and from all treated samples from passage 5 onwards (p<0.05). (C) The average number of the total colonies and total pluripotent-like colonies observed between passages 4–7. The number of pluripotent-like colonies in vehicle-treated cultures was significantly lower than that for all other treatments (p<0.05). None of the SB-216763-treated samples were significantly different from LIF-treated mESCs for either the total number of colonies or the total number of pluripotent-like colonies.

The role of β-catenin in mESC pluripotency and self-renewal is implicated by several genetics-based studies [13], [25], [32]. However, the ability of GSK3 inhibitors to maintain pluripotency for an extended period is less clear [32], [34]–[36]. Thus, we investigated whether SB-216763 could maintain J1 mESCs with an undifferentiated, pluripotent-like morphology in long-term culture. As expected, control mESCs maintained with LIF grew as compact colonies whose borders had high optical density and appeared as shadows and highlights on a Hoffman Modulation Contrast microscope or as glowing edges under a phase-contrast microscope (Figure 2A). These characteristics are widely accepted as a strong indicator of the pluripotent state (e.g., [44]). In a preliminary dose-response experiment, we found that LIF-free mESC medium and doses lower than 10 µM of SB-216763 were unable to maintain mESCs in a pluripotent-like state, whereas 30 µM SB-216763 yielded compact pluripotent-like colonies, which were much smaller and fewer in number (data not shown). This latter finding is consistent with observations of other groups that high doses of GSK3 inhibitors attenuate mESC proliferation [13], [32], [35]. Thus, to circumvent proliferation rate-related issues, we further investigated whether 10–20 µM SB-216763 could maintain mESCs in a pluripotent state.

J1 mESCs were cultured in LIF-free mESC medium or medium supplemented with LIF (1000 U/ml) or 10, 15 and 20 µM SB-216763. The cultures were passaged in parallel every 3–4 days for over a month. We observed that all doses of SB-216763 (10, 15, and 20 µM) and LIF maintained mESCs in a pluripotent-like state, and the mESCs cultured without any supplements (LIF-free) differentiated within two weeks (Figure 2A). MESC colonies in these samples were rated for pluripotency-like morphologies between passages 4 to 7 (weeks 2–4) (Figure 2B). Generally, we found that 80% of the colonies in 15 µM and 20 µM were rated as being pluripotent-like based on our criteria. LIF and 10 µM SB-216763 also maintained colonies in a pluripotent-like state, but there was less reliability in maintaining a high number of pluripotent-like mESC colonies between passages 4–7 in this experiment. As with 30 µM SB-216763 treatment, 20 µM SB-216763 yielded slightly fewer colonies which were compact and generally smaller than those seen with 10 µM SB-216763 (Figure 2C). Moreover, we observed that SB-216763 precipitated from the culture medium at doses ≥20 µM. The percentage of pluripotent-like colonies was not significantly different between LIF-treated mESCs and any of the SB-216763-treated samples (Figure 2C). For one of these follow-up experiments, the 10 µM SB-216763 and LIF treated cultures were continued for an additional month. At the end of the two month period, mESCs maintained with 10 µM SB-216763 were morphologically indistinguishable from those maintained with LIF (data not shown). These results suggest that SB-216763 can maintain mESCs with a pluripotent-like morphology in long-term culture.

### MESCs Maintained with SB-216763 for Greater than One Month Express Pluripotency-Promoting Genes at Levels Comparable to LIF-treated mESCs

**Figure 3:**
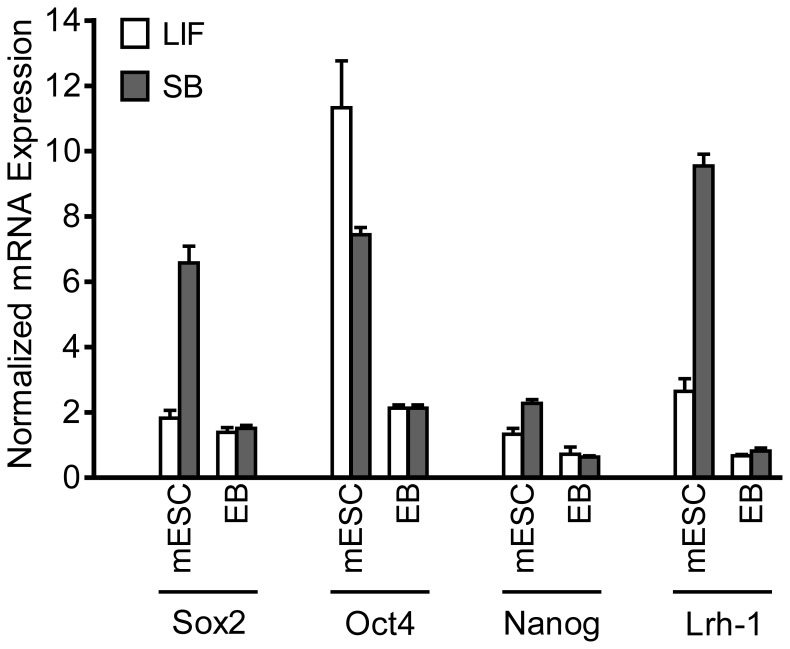
J1 mESCs maintained with 10 µM SB-216763 or LIF for more than a month express Oct4, Nanog, Sox2 and Lrh-1. The graph also shows expression of these markers in EBs derived from month-old LIF and SB-216763 mESCs. SYBR Green qPCR results for these four genes were normalized to β-actin housekeeping gene using qbasePLUS software. SB-216763 treated mESCs expressed Lrh-1 and Sox2 about 3-fold higher than LIF treated mESCs. Nanog expression was also moderately higher in the SB-216763-treated mESCs, whereas Oct-4 expression was nearly 50% higher in LIF-treated mESC. Expression of these genes in differentiated cells was generally lower than the mESCs maintained with either LIF or 10 µM SB-216763. The data are average ± S.D. of technical replicates from one of the two experiments.

We next determined the effects of long-term maintenance of mESCs with SB-216763 on the expression of pluripotency-specific genes. RNA transcripts for Oct4, Sox2, Nanog, and the β-catenin regulated gene Lrh-1, were quantified in mESCs maintained with LIF or 10 µM SB-216763 for one month [3], [7], [9]–[13]. We found that all four of these genes were expressed at high levels in both mESC cultures (Figure 3). However, expression of Sox2 and Lrh-1 was nearly 3-fold higher in SB-216763-treated mESCs compared to the LIF controls. Additionally, Nanog expression was moderately higher in SB-216763-treated cultures compared to the LIF control, whereas Oct4 expression was about 50% lower. Embryoid bodies (EB) prepared from these cultures after withdrawal of LIF or SB-216763 generally exhibited reduced expression of the pluripotency markers. These results suggest that the effects of SB-216763 on pluripotent-specific gene expression are reversible (Figure 3).

**Figure 4:**
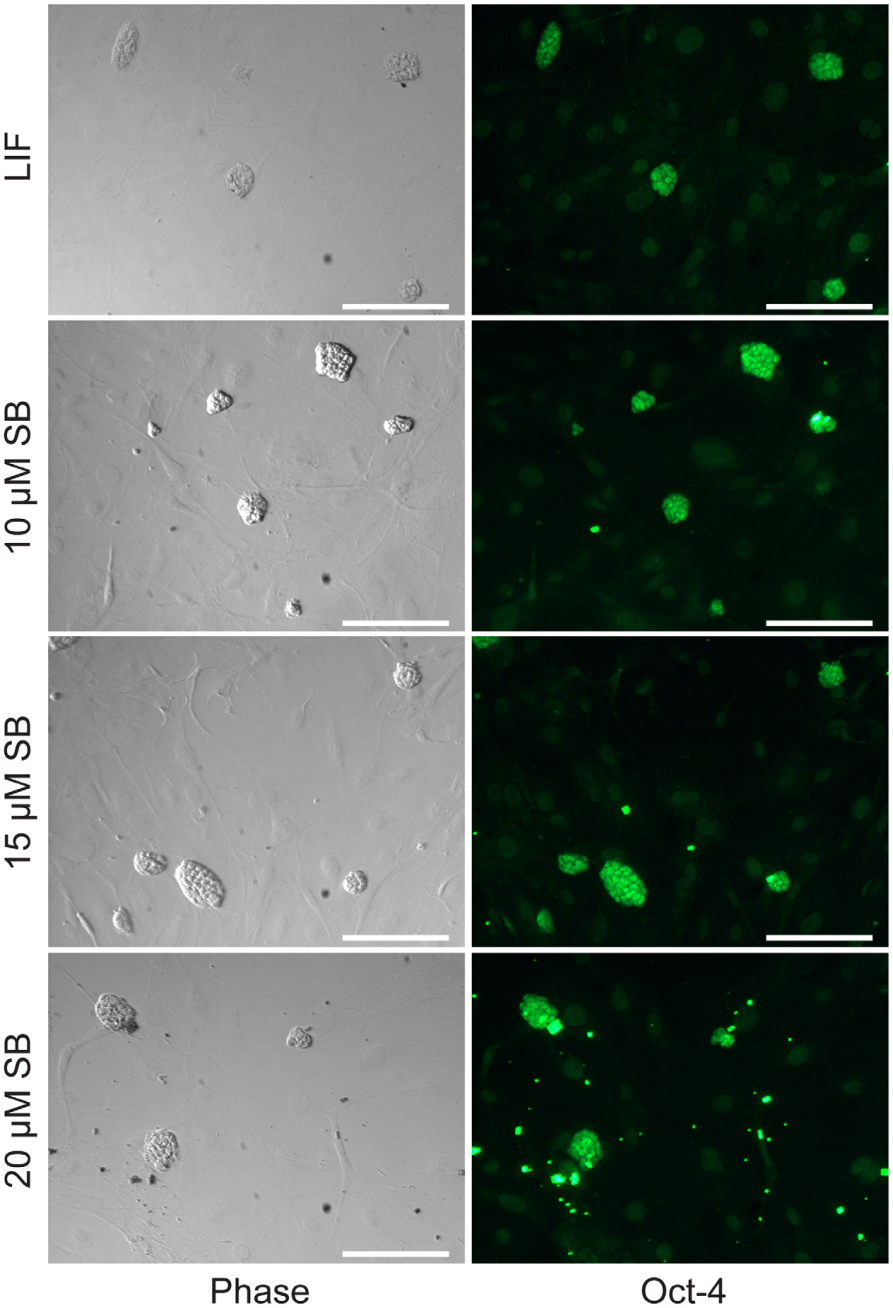
J1 mESCs maintained with SB-216763 for one month express the pluripotency marker Oct–4. Cultures were maintained continuously with various doses of SB-216763 or LIF for 1 month and then immunostained for Oct–4. SB-216763 precipitated at doses ≥20 µM, and some of these particulates are visible in the phase-contrast images as black spots that fluoresced intensely green when excited at ∼480 nm. Scale bars represent 100 µm.

**Figure 5:**
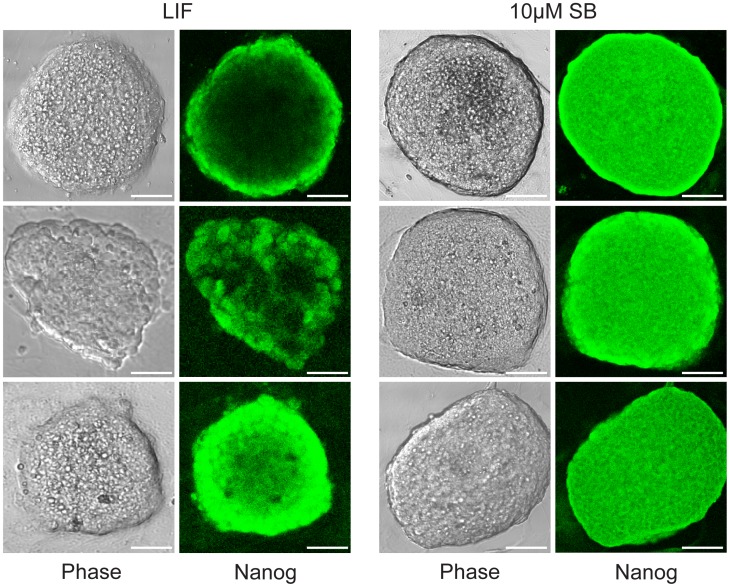
Expression of Nanog pluripotency marker is more homogeneous in J1 mESCs maintained with 10 µM SB-216763. MESCs maintained with either LIF or 10 µM SB-216763 for more than a month yielded pluripotent-like colonies. Immunostaining revealed that Nanog expression (green fluorescence) was homogeneous in mESC colonies maintained with SB-216763, whereas LIF-treated mESC colonies often displayed incomplete Nanog staining. Three representative colonies from each treatment are shown. Scale bars represent 40 µm.

Our qPCR results were subsequently confirmed by immunocytochemistry for Oct4 and Nanog, the two most widely used immunocytochemistry markers for pluripotent stem cells. LIF and all doses of SB-216763 (10, 15, and 20 µM) yielded compact mESC colonies that expressed Oct-4 (Figure 4). Detection of cellular Nanog was carried out on an independent culture that was grown for more than a month in the presence of LIF or 10 µM SB-216763. Surprisingly, Nanog immunostaining was distinctly more homogenous in the SB-216763-treated samples compared to the LIF-treated controls (Figure 5).

### SB-216763, Unlike BIO and CHIR-99021, can Maintain J1 mESCs in a Pluripotent State for More than a Month

**Figure 6:**
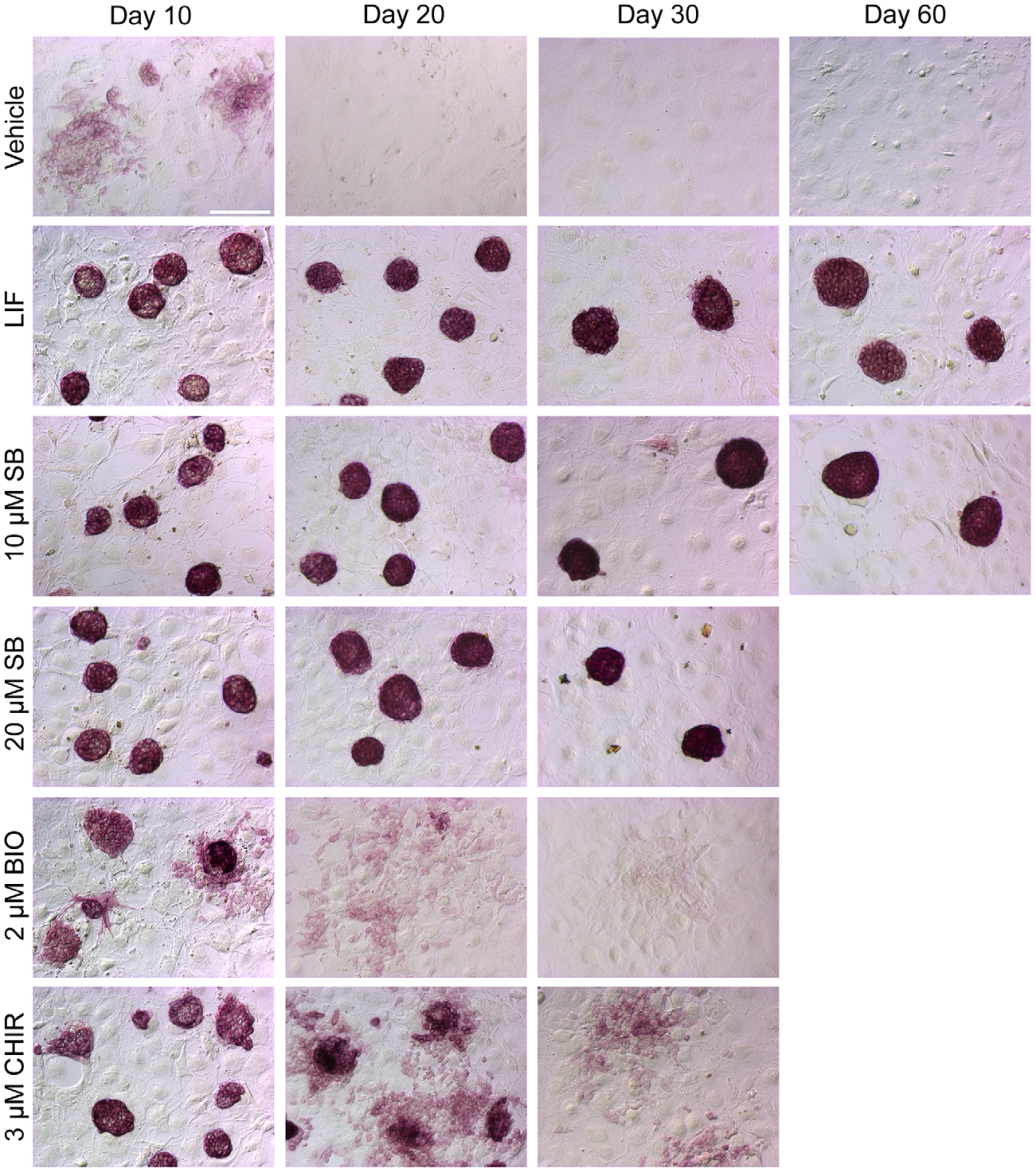
SB-216763, unlike BIO and CHIR-99021, can maintain J1 mESCs in a pluripotent state for at least two months. J1 mESCs co-cultured with MEFs were maintained continuously with vehicle (0.1% DMSO), 1,000 U/ml LIF, 10 or 20 µM SB-216763, 2 µM BIO, or 3 µM CHIR-99021. The cultures were passaged in parallel and 20,000–50,000 mESCs were plated on new MEF-covered 6-well plates every 3 days. A duplicate plate was stained for alkaline phosphatase (AP) activity at days 10, 20, and 30. Shown are representative images from one of two independent trials. Day 60 images were obtained from an independent culture maintained with vehicle (0.1% DMSO), 1,000 U/mL LIF, or 10 µM SB-216763. AP staining on day 60 was carried out on samples from a single trial. The scale bar shown in the day 10 vehicle image represents 100 µm and all images are presented at the same scale.

As shown previously, treatment of J1 mESCs for 24 hours under the conditions described here yielded TOPFlash reporter activity that was comparable between 2 µM BIO, 3 µM CHIR, and 10 µM SB-216763 treatments (Figure 1). Thus, we compared the ability of 2 µM BIO and 3 µM CHIR-99021 to sustain long-term cultures of J1 mESCs with parallel cultures maintained with 10–20 µM SB-216763 and LIF [34], [35]. The samples were stained for alkaline phosphatase (AP) activity, another well-known marker for pluripotent mESCs, on days 10, 20 and 30. As expected, vehicle treated samples differentiated by Day 10 and no colonies or AP positive cells were detected by day 20. Consistent with a previous report, BIO was able to maintain J1 mESCs with a pluripotent-like morphology for at least 10 days (Figure 6; [34]). By day 20, BIO samples had differentiated and after 30 days they were devoid of any pluripotent-like colonies. In general, 3 µM CHIR-99021 maintained pluripotent-like mESCs for a longer period of time compared to BIO, but most of the colonies were no longer tightly packed by day 20. After 30 days, the AP staining and colony morphology of CHIR-99021 samples resembled the differentiated BIO samples at day 20. In contrast, LIF and SB-216763 treated co-cultures yielded round, compact, AP-stained colonies at day 30 (Figure 6). An independent experiment further demonstrated that 10 µM SB-216763 can sustain AP-positive mESC colonies for at least up to 60 days (Figure 6; Day 60). To our knowledge, this makes SB-216763 the first GSK3 inhibitor that can maintain mESCs in a pluripotent state for at least two months.

### SB-216763 can Maintain MilliTrace Nanog GFP Reporter mESCs in a Pluripotent State

**Figure 7:**
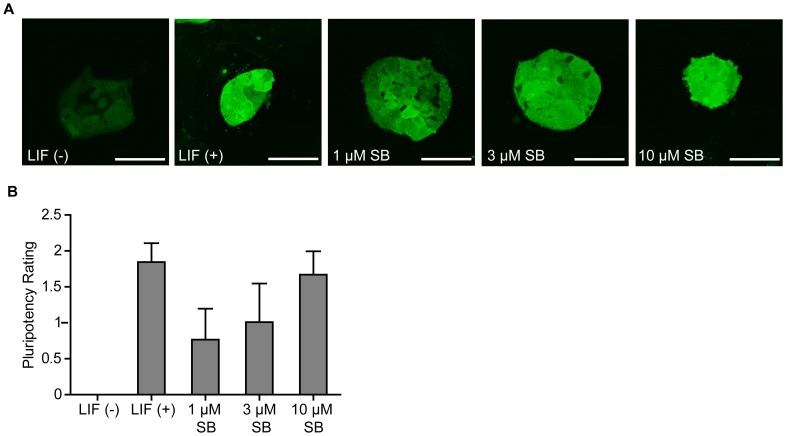
10 µM SB-216763 maintains MilliTrace Nanog GFP Reporter mESCs in a pluripotent-like state for more than a month. (A) GFP fluorescence in representative colonies from each treatment. (B) Mean pluripotency rating of two independent experiments ± SEM. Forty colonies were rated from each condition for the intensity and uniformity of GFP fluorescence after a month in culture. Statistical analysis revealed that the cultures maintained with LIF, 3 µM and 10 µM SB-216763 were significantly different than mESCs maintained without LIF.

To confirm that our results were not specific to J1 mESCs, we also maintained MilliTrace Nanog GFP Reporter mESCs with SB-216763 for one month. These mESCs express GFP under the control of the mouse Nanog promoter. Consistent with our expectations, MilliTrace mESCs maintained with 10 µM SB-216763 were morphologically indistinguishable from LIF-treated controls and exhibited robust GFP expression (Figure 7A). All the mESC colonies in LIF and 10 µM SB-216763 treated cultures expressed GFP at high levels and for the most part GFP distribution was homogeneous in these colonies. Scoring of GFP fluorescence intensity and homogeneous distribution of GFP showed that mESCs maintained with 10 µM SB-216763 were statistically not different from those maintained with LIF (Figure 7B). Some GFP expression was visible in nearly half of the mESC colonies grown without any supplements (LIF-free), but the expression was hardly ever uniform in these colonies. The LIF (-) panel in Figure 6A shows one of the GFP expressing colonies. We also observed a higher level of homogeneous GFP expression in the MilliTrace Nanog GFP Reporter mESCs maintained with 1 µM and 3 µM SB-216763 for more than a month. These results were unexpected because low doses of SB-216763 failed to promote pluripotent-like morphologies in the J1 mESCs (data not shown). It is possible that the MilliTrace Nanog GFP Reporter mESCs are more responsive to SB-216763, or that these mESCs are easier to maintain in a pluripotent state. As with Nanog immunostaining of J1 mESCs, the MilliTrace Nanog GFP Reporter mESCs maintained with 10 µM SB-216763 were more homogeneously labeled for GFP than the mESCs maintained with LIF or lower doses of SB-216763. Overall, these results suggest that 10 µM SB-216763 can maintain at least two independently-derived mESC lines in a pluripotent state for more than a month.

### MESCs Maintained with 10 µM SB-216763 can Differentiate into All Germ Layers

One of the defining characteristics of pluripotent cells is their ability to differentiate into all three lineages. Thus, we tested whether J1 mESCs maintained with SB-216763 retained their multi-differentiation potential by generating cardiac-like cells and neurons, and by monitoring expression of early stage markers of the three germ layers in EBs. MESCs maintained with 10 µM SB-216763 for more than a month differentiated into EBs as efficiently as those maintained with LIF. As shown in Figure 3, expression of pluripotency-promoting markers is lower in both EB samples, and indistinguishable from each other.

**Figure 8:**
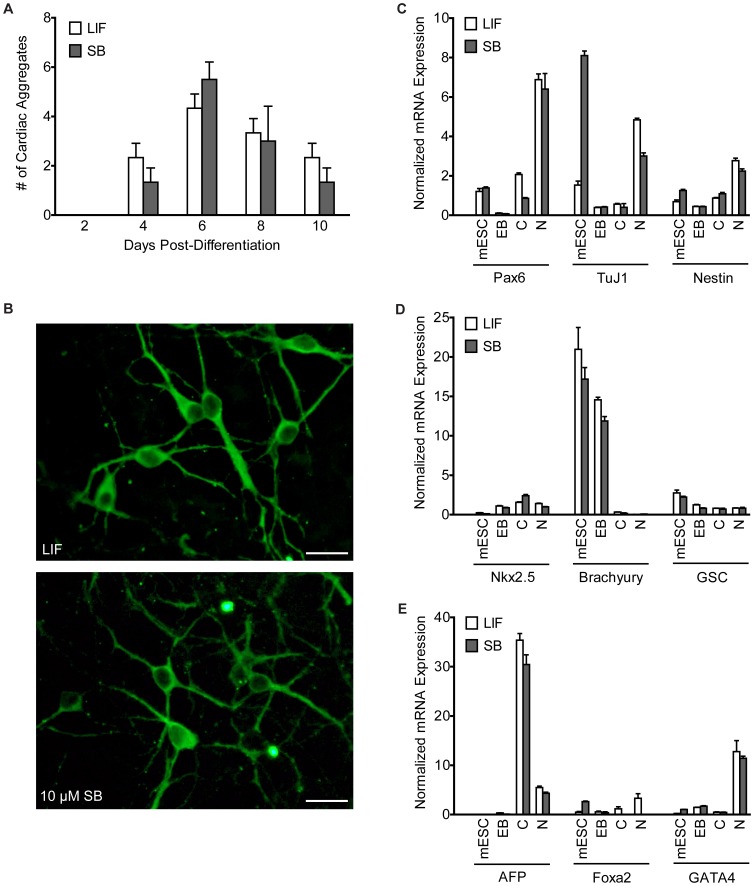
J1 mESCs maintained with SB-216763 retain their ability to differentiate into all three germ layers. Cultures maintained with 10 µM SB-216763 or LIF for more than one month were differentiated into (A) cardiac-like cells, (B) neurons, and EBs expressing transcript markers for (C) ectoderm or neuroectoderm, (D) mesoderm, and (E) endoderm. (A) The number of flattened autonomously contracting EBs was assessed every other day after the 4-day old EBs were transferred to a 24-well chamber (one EB/well). More than 25% of the 20 EBs analyzed per condition showed some level of autonomous contraction. (B) Neurons were stained for the neuronal marker gene MAP-2 by immunocytochemistry 16 days after EB formation. Scale bars represent 40 µm. (C-E) EBs prepared from mESCs maintained with either 10 µM SB-216763 or LIF conditions expressed early-stage transcript markers for all three germ layers. Data are representative from one experiment ± SD.

EBs derived from LIF- and SB-216763-supplemented mESCs readily differentiated into cardiac-like cells. The yield and appearance of cardiac-like cells were very similar between the two samples (Figure 8A). The autonomously contracting cells within EBs were first detected 4 days after EB attachment, which peaked on day 6 and gradually decreased thereafter (Figure 8A). Furthermore, the cardiac-like cells derived from these two mESC cultures appeared to be similar for EB size, contracting cluster size and the frequency of contractions (data not shown).

EBs differentiated into neurons using all-trans retinoic acid and the 4−/4+ protocol also yielded similar morphologies and expression of MAP-2 (a marker of mature neurons) [38]. Neurites were apparent two days after attachment (10 days after EB formation) and neurite arborization increased for another week. Immunocytochemistry for MAP-2 18 days after EB formation showed that the yield of neurons and their morphology were not dependent on the supplement used to maintain mESC in a pluripotent state (Figure 8B).

We also analyzed expression of the early-stage markers for the three germ layers in EBs, cardiac-like cells and neuronal cells. Our results show that expression of all the lineage-specific genes is comparable within each respective cell type regardless of whether the mESCs were maintained with LIF or 10 µM SB-216763. The ectodermal/neuroectodermal markers, Nestin and Pax6, were expressed at the highest levels in the neuronal sample (8 days after EB formation) and their expression levels were comparable between neurons derived from mESCs maintained with LIF and SB-216763 (Figure 8C). Expression of Tuj1 was nearly four-fold higher in pluripotent mESCs maintained with SB-216763 compared to mESCs maintained with LIF (Figure 8C). This pattern of Tuj1 expression may be expected based on the previous report that TWS119, a GSK3 inhibitor, can direct differentiation of mESCs into neurons without EB formation [45].

Quantification of three mesodermal markers – Nkx2.5, Brachyury, and Goosecoid (GSC) – in mESCs, EBs, cardiac-like and neuronal cells showed only minor differences between the two treatments. Regardless of the pluripotency maintenance supplement, Brachyury expression was the highest during mESC and EB stages. The high level of Brachyury expression in mESCs was a surprising result. Brachyury is an early stage marker of mesoderm, and its increased expression in predominantly pluripotent mESC cultures possibly suggests that spontaneously differentiating cells in the culture are following the normal process at these initial stages [46]. Nkx2.5 expression was relatively low but increased modestly from mESCs to EBs to cardiac-like cells regardless of whether the mESCs were maintained in LIF or SB-216763 (Figure 8D).

Among the endodermal markers, alpha-fetoprotein (AFP) was expressed at the highest level in cardiac-like cells, and at modest levels in neurons (Figure 8E). We also observed high levels of GATA4 in both neuronal samples (Figure 8E). This may support the presence of astrocyte precursors and or specific neuronal precursors [47], [48]. Foxa2 expression was very low in all cell types (Figure 8E). A comparison of samples derived from LIF- and SB-216763-treated mESCs revealed only minor differences in expression levels of these three endodermal markers.

These results show that mESCs maintained in 10 µM SB-216763 for more than a month can successfully differentiate into cardiac-like cells and neurons, and can express germ line markers in the differentiated cells at levels comparable to LIF-maintained mESCs.

## Discussion

The canonical Wnt pathway is essential for the development of all animals [18], [49], [50]. In bilaterians, canonical Wnts usually lead to sequestration of GSK3 and accumulation of β-catenin, which in a context-dependent manner signals for proliferation, cell lineage specification, and maintenance of the pluripotent and multipotent states [15]–[18], [51]. Thus, several groups have tested whether small molecules that activate the canonical Wnt pathway and increase β-catenin-mediated gene expression, can maintain stem cells in a pluripotent state [32], [34]–[36]. Here, we show that 10 µM SB-216763, a GSK3 inhibitor, can maintain J1 and MilliTrace Nanog GFP Reporter mESC lines in a pluripotent state for more than a month. We have used four assays to support this conclusion: (1) colony morphology, (2) expression of pluripotency markers and pluripotency-associated genes, (3) effective differentiation into cardiac-like and neuronal cells, and (4) expression of early-stage markers in EBs representing all three germ layers. MESCs maintained with 10 µM SB-216763 were indistinguishable or slightly better than LIF treated mESCs for each of these assays.

The concentration of SB-216763 needed for optimal maintenance of pluripotency in J1 mESCs also yielded robust activation of the β-catenin reporter gene. In mESCs, β-catenin is thought to mediate its actions through both its functions at the adherens junction as well as the nucleus [32], [52]–[54]. One of the well characterized functions of β-catenin in the nucleus is its ability to alleviate TCF3’s repression of pluripotency promoting genes [12], [26]. Chromatin-immunoprecipitation assays have shown that the vast majority of TCF3 target promoters are also occupied by Oct4, Sox2 and Nanog [26], [27]. Deletion of TCF3 increases levels of Oct4 and Nanog, and also eliminates the need for Wnt3a or GSK3 inhibition for maintenance of self-renewal [26], [27]. One of the direct targets of this de-repression is Lrh-1 [13]. Expression of Lrh-1 appears to be dependent on β-catenin, and in turn Lrh-1 can promote expression of pluripotency transcription factors Oct-4 and Nanog [13]. Consistent with this, we found that long-term maintenance of mESCs in a pluripotent state with 10 µM SB-216763 induces higher levels of Lrh-1 compared to the parallel mESC cultures maintained with LIF (Figure 5). These observations support the idea that the β-catenin/Lrh-1 signaling axis is important for maintenance of pluripotency by β-catenin activators such as SB-216763.

Several lines of genetic evidence suggest that β-catenin is an important determinant of self-renewal and maintenance of the pluripotent state [13], [25], [32], [33], [54]. Thus, small molecule inhibitors of GSK3 that block degradation of β-catenin should be able to promote pluripotency. The earliest evidence that a GSK3 inhibitor could promote pluripotency was demonstrated with BIO. BIO induced expression of Oct-4, Rex-1 and Nanog in both human and mouse ESCs and supported pluripotency for at least 1 week [34]. However, these effects cannot be considered GSK3-specific because the doses used for BIO were nearly 1000-fold higher than BIO’s IC_50_ for GSK3 of 5nM and were approximately 10-fold higher than BIO’s IC50 for other kinases such as CDK1, CDK2, and CDK5 [55]. Moreover, two other groups have suggested that these effects are short-lived and not suitable for long-term maintenance of stem cells in a pluripotent state [13], [35]. More selective GSK3 inhibitors such as CHIR-99021 and bisindolylmaleimides also fail to maintain mESCs in a pluripotent state for the long-term by themselves [32], [34]–[36]. We initially tested SB-216763 because it is a fairly selective and potent inhibitor of GSK3. For example, in an in vitro assay, 10 µM SB-216763 reduced β-catenin activity to <5% without dramatically affecting 24 other kinases tested [56]. This raises the possibility that SB-216763 may be mediating some of its pluripotency promoting effects through other kinases. CHIR-99021 and SB-216763 belong to different classes of compounds and therefore these drugs bind to different sets of nonspecific targets with varying affinities; CHIR-99021 is an aminopyrimidine whereas SB-216763 is an arylindolemaleimide [41], [56]–[58]. Comparison of these two compounds for their effect on a panel of kinases showed that CHIR-99021 is more selective than SB-216763, but both compounds also inhibit other kinases: 1 µM CHIR-99021 can reduce activity of CDK2, PLK1 and MELK by >50%, while 10 µM SB-216763 reduces activity of CDK2, ERK8, DRYK1A, PIM3, SRPK1, HIPK2 and HIPK3 by >50% [41]. Our prediction is that the off-target effects of SB-216763 contribute to pluripotency maintenance, while the off-target effects of BIO, CHIR-99021 and bisindolylmaleimides do not. However, there is no evidence to support the idea that these off-target effects are more important than the GSK3 inhibition (e.g., Figure 6). We therefore suggest that SB-216763’s off-target effects work synergistically with GSK3 inhibition to promote pluripotency. For example, it is possible that inhibition of homeodomain-interacting protein kinase 2 (HIPK2) could enhance SB-216763’s ability to maintain pluripotency. HIPK2 can phosphorylate β-catenin and target it for degradation in a manner similar to GSK3 [59]. In the case of mouse epidermal stem cells, loss of HIPK2 results in an increase in cell proliferation rate [60]. Thus, SB-216763 mediated inhibition of HIPK2 (85% inhibition at 10 µM [41]) could work synergistically with GSK3 inhibition. Pharmacological evaluation of this GSK3/HIPK2 dual inhibition hypothesis cannot be explored right now as HIPK2 specific inhibitors are not yet commercially available.

There is an added level of complexity with GSK3 inhibitors and GSK3 mutants. GSK3α and GSK3β are integral components of several pathways and attenuation of GSK3 function affects all of those pathways [61], [62]. From these GSK3-associated signaling pathways, the β-catenin pathway is considered to be most important for the maintenance of pluripotency [13], [32]. Consistently, our results demonstrate that the SB-216763 concentrations needed for optimal growth and self-renewal of pluripotent mESCs robustly activate β-catenin reporter gene in mESCs (Figure 1). However, our results also show that activation of β-catenin alone is not sufficient for maintenance of pluripotency (Figures 1 and 6). As discussed above, we recognize that almost all small molecules have off target effects and that some of these other targets could also contribute to SB-216763’s ability to maintain mESCs in a pluripotent state. Further evaluation of the role of these other SB-216763 target proteins in maintenance of pluripotency can enhance our knowledge of the signal transduction pathways that regulate cell fate decisions. Nonetheless, our results unequivocally demonstrate that SB-216763 can maintain mESCs co-cultured with MEFs in a pluripotent state for up to two months in the absence of exogenous LIF.

## Supporting Information

Table S1 Primer pairs used for mESC and germ line markers.
(TIF)
