# Supplementary figures and images for: Glycogen Synthase Kinase 3 (GSK3) Inhibitor, SB-216763, Promotes Pluripotency in Mouse Embryonic Stem Cells
Source: PLoS One. 2012 Jun 26;7(6):e39329. doi: 10.1371/journal.pone.0039329 (PMC3383737; doi:10.1371/journal.pone.0039329)

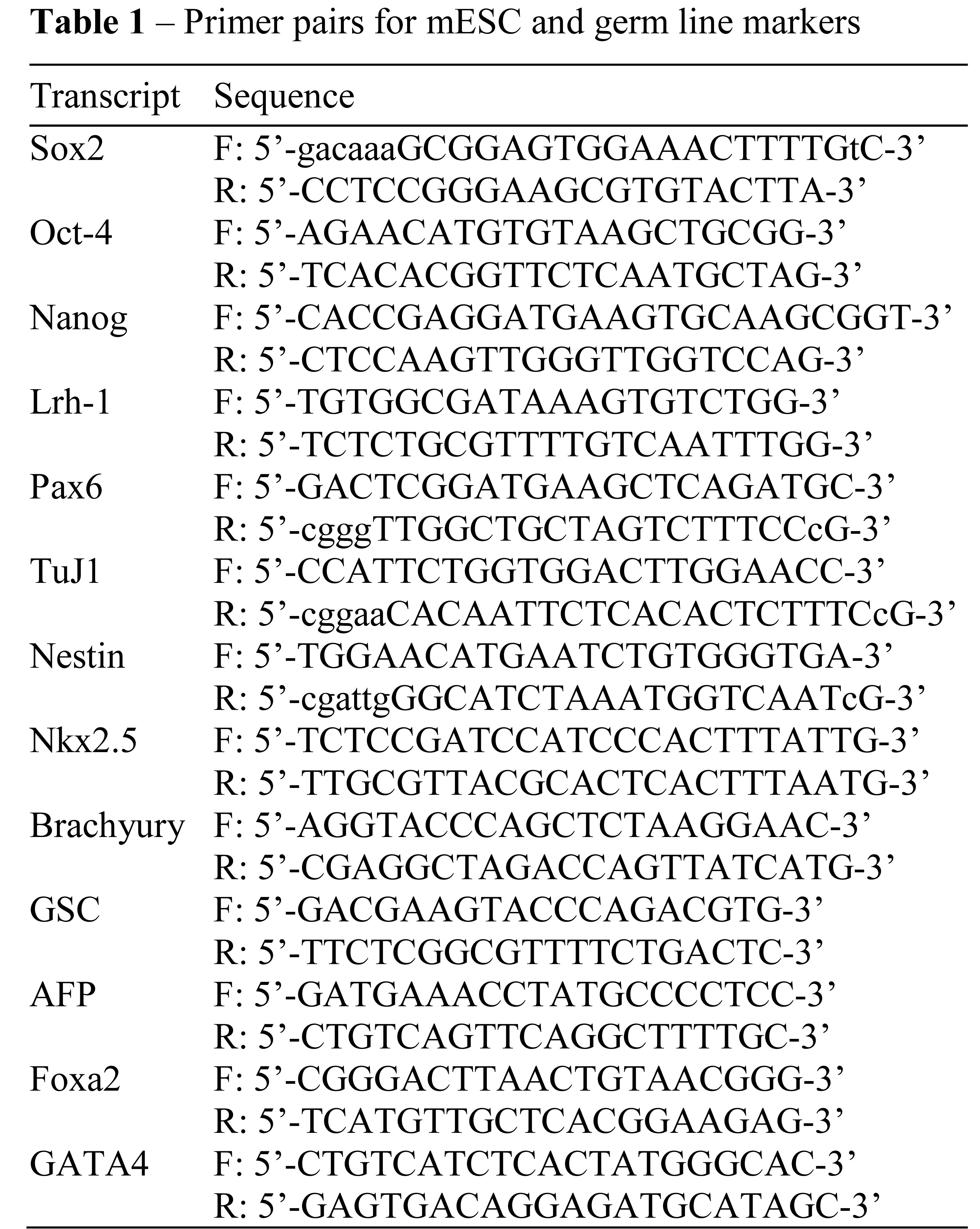

Supplement: Table S1 — Primer pairs used for mESC and germ line markers. (TIF) [file pone.0039329.s001.tif]
